# Prediction of Immune Checkpoint Inhibitor-Induced Liver Injury in Patients with Gastrointestinal Cancer: Machine Learning Modeling for Time-Stratified Risk Assessment

**DOI:** 10.3390/curroncol33090568

**Published:** 2026-09-20

**Authors:** Ying Jiang, Ranyi Li, Hong Gao, Xiaoyu Li, Ningping Zhang

**Affiliations:** 1 Department of Pharmacy, Zhongshan Hospital, Fudan University, 180 Fenglin Road, Shanghai 200032, China; jiang.ying5@zs-hospital.sh.cn (Y.J.); li.ranyi@zs-hospital.sh.cn (R.L.); 2 Department of Gastroenterology, Zhongshan Hospital, Fudan University, Shanghai 200032, China; gao.hong@zs-hospital.sh.cn

**Keywords:** machine learning, immune checkpoint inhibitors, irAE, gastrointestinal cancer, SHAP

## Abstract

**Background:** Immunotherapy has transformed cancer treatment and is widely used in the Chinese mainland. Though advances have been made, immune checkpoint inhibitor-related liver injury (ICILI) remains a significant clinical challenge. Existing risk models commonly lack time-specific risk stratification for ICILI. The present study aimed to develop and validate interpretable machine learning models to predict grade 2 or higher ICILI at multiple time points in patients with gastrointestinal cancer (GC). **Methods:** This retrospective cohort study encompassed GC patients who commenced their initial ICI medication between January 2019 and June 2023 at Zhongshan Hospital, Fudan University. Five machine learning algorithms, including Logistic Regression, Random Forest, Extreme Gradient Boosting (XGBoost), Gradient Boosting (GradientBoost), and Adaptive Boosting (AdaBoost), were utilized to develop predictive models for grade ≥ 2 ICILI at specific intervals of 3 months, 6 months, and 12 months. The evaluation of model performance was conducted using the area under the curve (AUC), accuracy, precision, recall, and F1-score. The Shapley Additive exPlanations (SHAP) method was employed to assess feature importance and interpret the final model. **Results:** A total of 1337, 849, and 401 patients were enrolled in the follow-up groups at 3 months, 6 months, and 12 months. The final model for grade ≥ 2 ICILI was developed with GradientBoost and achieved an AUC of 0.769 (95% CI: 0.732–0.806), with a test set accuracy of 0.834. XGBoost yielded AUCs of 0.671 (95% CI: 0.636–0.706) at 3-month indication, 0.678 (95% CI: 0.638–0.718) at 6 months, and 0.644 (95% CI: 0.589–0.699) at 12-month follow-up in the 5-fold cross-validation. The DCA curve demonstrated solid clinical benefit, whereas the calibration curve indicated good predictive reliability. SHAP analysis identified several parameters as predictive features at different intervals, which suggested that the ICILI determinants varied from acute inflammatory to host-related characteristics. **Conclusions:** A temporal stratification prediction model for grade ≥ 2 ICILI in GC patients was developed and validated at various intervals using ML algorithms with SHAP interpretability. This methodology facilitated early recognition of varying parameters across different treatment phases, enhancing clinical management and elevating treatment outcomes.

## Simple Summary

Immune checkpoint inhibitors are widely used in the treatment of gastrointestinal cancers. Yet, immune-related liver injury continues to be a notable unfavorable occurrence for which dependable prediction tools are lacking. Current models provide single-point risk assessments and fail to consider the evolving characteristics of risk during therapy. In this research, time-stratified machine learning models were developed using data from 1337 patients to predict liver injury at 3, 6, and 12 months following therapy initiation. The GradientBoost model exhibited good prediction efficacy; however, SHAP analysis identified a temporal alteration in prevailing risk factors. Initial inflammatory indicators transitioned to host characteristics, encompassing gender and body mass index. These temporal models enable clinicians to identify changing risk profiles and implement specific monitoring strategies throughout various phases of treatment. This approach provides a progression toward individualized and enhanced safety in immunotherapy administration.

## 1. Introduction

In recent decades, malignancies originating from the gastrointestinal tract, such as oesophagal, gastric, colonic, and rectal cancers, have appeared as major contributors to the worldwide cancer burden, alongside an extensive economic burden. In various regions of South and Central Asia, GI cancers (GC) continue to be a predominant cause of cancer mortality [1,2]. Despite multimodal treatment strategies, achieving durable disease management in individuals with advanced or treatment-resistant GC remains a significant clinical challenge. Yet, immune checkpoint inhibitors (ICIs) targeting programmed cell death-1 (PD-1) and programmed death-ligand 1 (PD-L1) have shown major improvements in clinical survival rates for patients with refractory GC in various phase II-III randomized trials, illustrating a significant advancement in oncological therapy [3,4,5].

The emergence of immunotherapy has transformed cancer therapy. However, the extensive enhancement of immune responses due to ICI interference with checkpoint signaling might lead to undesirable effects known as immune-related adverse events (irAEs) [6]. ICI-associated liver injury (ICILI) is a common and potentially severe complication, with reported incidence rates ranging from 1% to 25% [7,8,9]. To date, the majority of current research has focused on the clinical characteristics of ICILI in pan-cancer or hepatocellular carcinoma (HCC) populations [10,11], resulting in a notable deficiency in the cohort of GC. However, evidence has also shown that ICIs, as part of immunotherapy for GC, have led to a rise in ICILI cases, some of which even progress to liver failure [12,13]. Hence, further research is necessary to facilitate early identification of ICILI in patients with GC, particularly by developing early-warning strategies and reliable prediction models to enhance the clinical effectiveness of ICI-based treatment.

At present, there are no predictive models that particularly target ICILI in patients with GC. Numerous investigations have identified possible risk factors for ICILI in various cancer types, such as female gender, reduced baseline alkaline phosphatase (ALP), elevated alanine aminotransferase (ALT), HCC and hepatitis B [14,15], few practical prediction tools have been developed. For example, Jun et al. developed a logistic regression model to predict ICI-associated hepatotoxicity, achieving an area under the receiver operating characteristic curve (AUROC) of 0.752 [16]. Zheng et al. constructed a nomogram-style logistic analysis to predict sintilimab-related ICILI, obtaining a C-index of 0.713 and an AUC of 0.563 [17]. While these traditional statistical models are valued for their simplicity and interpretability, their ability to incorporate many indicators is limited, mostly due to intrinsic challenges in expressing nonlinear associations and complex interactions among variables [11]. Thus, machine learning (ML) algorithms have attracted significant interest as a powerful option due to their superior capacity to analyze different datasets and detect interactions among factors.

This study attempted to develop and validate an ML-based model with real clinical data to incorporate clinical variables. Numerous ML models were developed to predict the likelihood of ICILI in patients with GC after ICI treatment at different intervals. Moreover, approaches for model interpretability were employed to identify the predictive factors, thus providing a reference for risk evaluation in immunotherapy.

## 2. Materials and Methods

### 2.1. Study Design and Patient Population

A retrospective cohort study was conducted to investigate the clinical characteristics and develop predictive models for ICILI in GC patients at Zhongshan Hospital, Fudan University, from January 2019 to June 2023. The study included individuals aged 18 and above who underwent their initial administration of PD-1 or PD-L1 inhibitor treatment and experienced at least three months of follow-up. Patients were excluded from the study if any of the subsequent conditions arose between the commencement of ICIs and the evaluation of ICILI: (1) involvement in clinical trials with undisclosed data; (2) elevated liver enzymes attributed to medications other than ICIs or advancement of liver metastasis; (3) hospitalization with a diagnosis of sepsis, cardiac failure, obstructive jaundice, or autoimmune liver disease; (4) inadequate baseline liver function test results acquired prior to or following the initial ICI treatment; or (5) lack of follow-up liver function tests performed three months after the first ICI therapy. The ethical committee of Zhongshan Hospital, Fudan University, approved this research (B2025-334R) on 7 July 2025, and the requirement for informed consent was waived.

For time analyzes, we established three distinct follow-up cohorts dependent on data availability at each specified time point: the 3-month cohort (patients with complete liver function data at 3 months, *n* = 1337), the 6-month cohort (patients with complete data at 6 months, *n* = 849), and the 12-month cohort (patients with complete data at 12 months, *n* = 401). These signify separate cross-sectional groups at each interval, and patients without follow-up data at a specific period were excluded from the relevant study.

### 2.2. Outcomes and Definitions

The identification of ICILI was established in accordance with the 2023 Chinese guidelines for the diagnosis and management of drug-induced liver injury (DILI) [18]. Based on the established exclusion criteria, two clinical pharmacists (Y.J and R.L) excluded patients with elevated liver function due to diseases such as progression of liver metastases, reactivation of hepatitis B virus, autoimmune liver disease, or hepatotoxicity that was considered to be caused by other medications by assessing consecutive liver function examinations, abdominal imaging investigations, and medication records. The suspected cases were evaluated in detail and subsequently resubmitted to the hepatologist (N.Z) for further confirmation. The Roussel Uclaf causality assessment method (RUCAM) was used to assess the relationship between ICI therapy and the possible liver injury, with a RUCAM score of 3 or higher classified as the ICILI group. Total RUCAM scores can be interpreted as follows: 0 point, drug is ‘excluded’ as a cause; 1~2, ‘unlikely’; 3~5, ‘possible’; 6~8, ‘probable’; and >8, ‘highly probable.’ To improve diagnostic validity, a sensitivity analysis limited to RUCAM ≥ 5 was performed to evaluate the reliability of the model predictions in relation to diagnostic uncertainty.

The primary objective centered on the emergence of grade ≥ 2 ICILI subsequent to ICI therapy, with severity classified into 5 levels according to the Common Terminology Criteria for Adverse Events (CTCAE, version 5.0) [19]. The secondary endpoint was the incidence of grade ≥ 2 ICILI at each time-specified cohort, specifically 3 months, 6 months, and 12 months following the commencement of treatment.

### 2.3. Data Extraction and Processing

A thorough review of electronic medical records was performed to gather information on clinical characteristics, laboratory parameters, and medications. Variables exhibiting over 50% missing data were eliminated to reduce bias, while the residual missing values were filled in Light Gradient Boosting Machines. Due to the intrinsic scarcity of grade ≥ 2 ICILI occurrences, leading to a significant class imbalance in the training set, the Synthetic Minority Oversampling Technique (SMOTE) was utilized to enhance the minority class. Before modeling, all continuous variables underwent Z-score standardization, and categorical factors were converted using ordinal numerical encoding [20].

### 2.4. Feature Selection and Development of Prediction Models

The complete group was randomly divided into a training set and an internal validation set at an 8:2 proportion. The feature selection approach integrating Random Forest and Least Absolute Shrinkage and Selection Operator (LASSO) was employed in the training set to achieve equilibrium and reduce model complexity. In the Random Forest classifier, variable importance was evaluated in Gini impurity for each predictor across all decision trees in the ensemble. Meanwhile, LASSO facilitated dimensionality reduction by adjusting the penalty parameter lambda (λ), which progressively reduces the coefficients of non-essential or redundant predictors to zero [20]. From both methodologies, we identified the ten most significant features and selected the combined feature set as candidate variables for ML models.

We developed five machine learning prediction models using the training set: Logistic Regression, Random Forest, XGBoost, Gradient Boosting Classifier, and AdaBoost. Hyperparameter optimization for each model was performed through grid search combined with 5-fold cross-validation to identify optimal parameter settings.

### 2.5. Model Evaluation and Interpretation

We assessed discrimination and classification performance using the area under the receiver operating characteristic curve (AUC), accuracy, recall (sensitivity), specificity, and F1-score. 95% confidence intervals (CI) were obtained with the bootstrap method with 1000 resampling repetitions. Calibration plots were established to visually assess the alignment between predicted probability and actual occurrence frequencies. In addition, decision curve analysis (DCA) was conducted to identify clinically meaningful thresholds. Finally, we performed an interpretability analysis of the final optimized model with the SHapley Additive exPlanations (SHAP) method.

### 2.6. Statistical Analysis

Baseline clinical characteristics were compared between GC patients who experienced ICILI and those who did not. We initially used the Shapiro–Wilk test for all continuous variables to assess normality. Continuous data that followed a normal distribution were exhibited as mean ± standard deviation, while non-normally distributed variables were represented as median with interquartile range. Categorical variables were expressed as numbers (%). Student’s *t*-test or Mann–Whitney U-test were used in the analysis of continuous variables, whereas categorical variables were evaluated through the Chi-squared test or Fisher’s exact test, with a two-tailed *p*-value lower than 0.05 deemed statistically significant. Statistical analyzes were performed using Python 3.12.3 and R version 4.3.

## 3. Results

### 3.1. Study Cohort and Patient Characteristics

**Figure 1:**
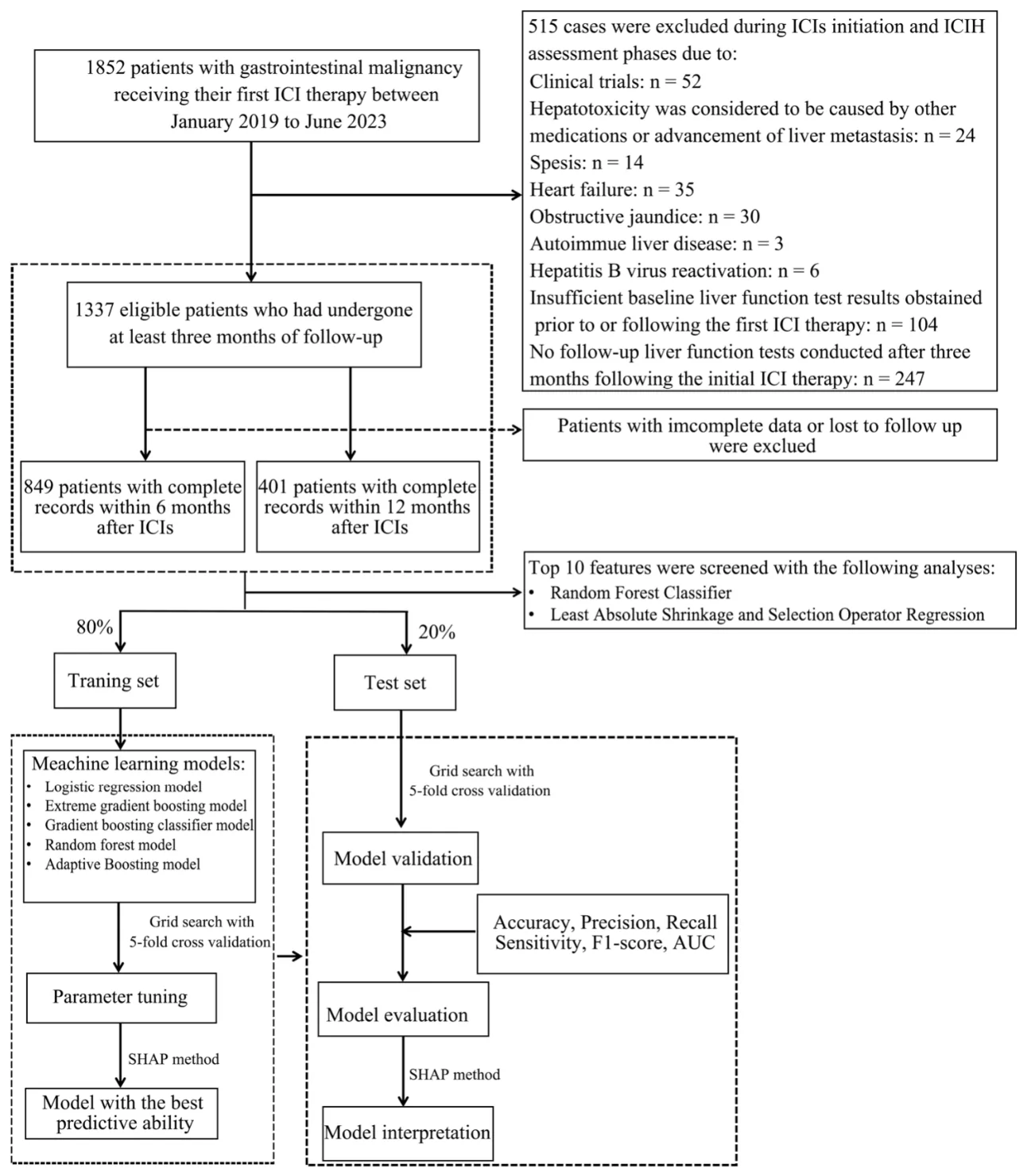
Flow chart of patient selection and study design. ICI: immune checkpoint inhibitor; ICILI: Immune checkpoint inhibitor-associated liver injury; AUC: area under the receiver operating characteristic curve; SHAP: Shapley Additive exPlanations.

As shown in Figure 1, we initially recruited 1852 patients with GC who commenced ICI therapy between January 2019 and June 2023. After excluding patients participating in clinical trials (*n* = 52), individuals with elevated liver enzymes were attributed to hepatotoxicity from alternative medications or the advancement of liver metastasis (*n* = 24), sepsis (*n* = 14), heart failure (*n* = 35), obstructive jaundice (*n* = 30), autoimmune liver disease (*n* = 9), incomplete baseline liver function data (*n* = 104), and those lacking follow-up liver function records within 3 months of ICI treatment (*n* = 247), the final cohorts with complete follow-up at 3, 6, and 12 months comprised 1337, 849, and 401 patients, respectively. The incidence proportions of grade ≥ 2 ICILI within the 3-, 6-, and 12-month cohorts were 8.38% (112/1337), 14.15% (106/849), and 11.97% (48/401) respectively (Table 1). Of these, 62, 54, 53 and 12 were classified as grade 1 to 4 (Supplementary Table S1).

At all follow-up intervals, patients who experienced ICILI exhibited a higher incidence of liver metastases in comparison to those without ICILI (3 months: 46.4% vs. 27.6%; 6 months: 49.1% vs. 29.3%; 12 months: 45.8% vs. 29.2%; *p* < 0.05) and elevated baseline levels of ALT, aspartate aminotransferase (AST), and C-reactive protein (CRP) (*p* < 0.05). Analysis of hepatotoxicity severity (Supplementary Table S1) showed that the occurrence of liver metastases, along with the levels of AST, ALT, gamma-glutamyl transferase (GGT), CRP, carcinoembryonic antigen (CEA) and carbohydrate antigen 19-9 (CA19-9), escalated with greater severity grade (*p* < 0.05). Moreover, HBV infection and in combination with tyrosine kinase inhibitors (TKIs), was more prevalent in cases of elevated ICILI severity (*p* < 0.05). The data suggested that liver metastases, elevated liver enzyme levels, baseline inflammatory markers, and concurrent TKI administration are pivotal risk factors for the development and progression of ICILI.

Given the significant reduction in sample size as follow-up advanced, we analyzed baseline characteristics between patients who continued in follow-up and those who were censored at each interval to evaluate possible bias. Supplementary Table S2 showed that many variables, such as CRP, liver function assessments, tumor burden metrics, and concomitant conditions, did not differ significantly between the retained and censored groups at 6 or 12 months. The only significant distinction was a higher rate of concurrent TKI use in the 12-month retained group (7.0% vs. 3.4%, *p* < 0.001), which may be attributable to temporal shifts in treatment regimens. These results indicate that the variations in feature significance are not mostly influenced by survivor bias.

### 3.2. Feature Selection and Model Development

Feature selection analysis at specific time points revealed alterations in the variables closely associated with ICILI risk. For grade ≥ 2 ICILI (Supplementary Figure S1A–C), the primary factors were C-reactive protein, liver metastases, and platinum-based treatment. In the initial 3 months of immunotherapy (Supplementary Figure S1D–F), indicators of acute liver injury, such as AST and ALT, as well as abnormal coagulation, became prominent. At the 6-month point (Supplementary Figure S1G–I), cardiovascular markers like B-type Natriuretic Peptide (BNP) were significantly influential. At the 12-month follow-up, the model identified parameters linked to significant tumor metastasis (≥3 metastatic sites), baseline liver function (ALB or GGT), and underlying diseases (Supplementary Figure S1J–L). The union for the final predictors at specific time cohorts were exhibited in Supplementary Table S3.

### 3.3. Final Model Construction and Evaluation

**Figure 2:**
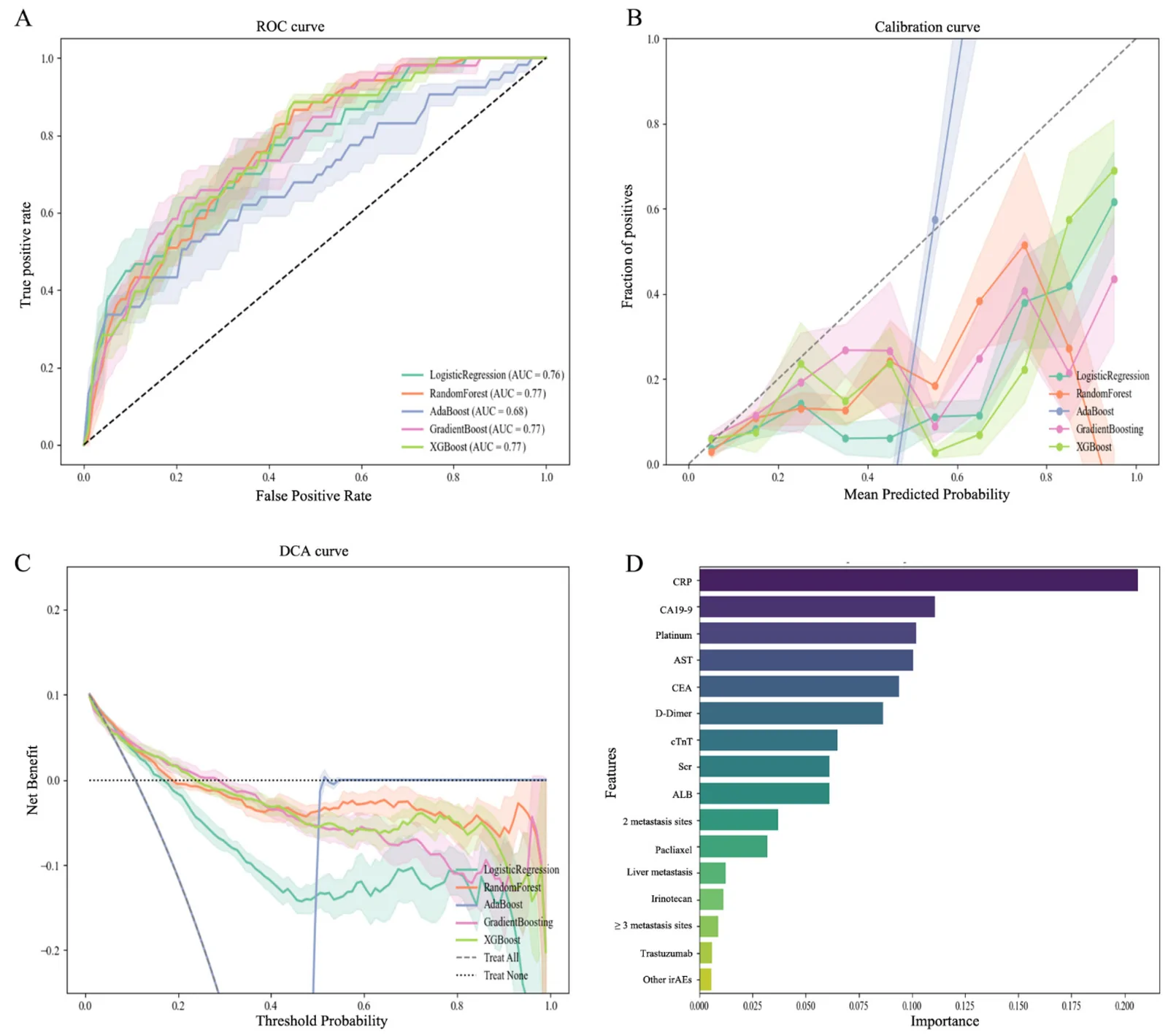
Performance of Machine Learning for gastrointestinal cancer patients with ICILI ≥ grade 2. (**A**–**C**) ROC, calibration curves and DCA for different models. (**D**) Feature importance in the Gradient Boost model. ICILI: Immune checkpoint inhibitor-associated liver injury; AUC: area under the receiver operating characteristic curve; ROC: receiver operating characteristic curve; DCA: decision curve analysis; CRP: C-reactive protein; CA19-9: carbohydrate antigen 19-9; AST: aspartate aminotransferase; CEA: carcinoembryonic antigen; cTnT: cardiac troponin T; Scr: serum creatinine; ALB: albumin; irAEs: immune-related adverse events.

We developed and assessed ML algorithms to predict grade ≥ 2 ICILI in individuals with GC, the details of which were provided in Table 2 and the corresponding performance curves were shown in Figure 2. GradientBoost achieved the highest AUC of 0.769 (97% CI: 0.732, 0.806) for prediction of grade ≥ 2 ICILI. The calibration curve exhibited a good correlation with the optimum diagonal (Figure 2A,B). Decision curve analysis showed that GradientBoost provided greater net clinical benefit than alternative models within the threshold probability range of 0.1 to 0.6 (Figure 2C). Consequently, GradientBoost was chosen as the principal model for the comprehensive prediction assignment.

For the 3-month prediction, the XGBoost model got an AUC of 0.671 (95% CI: 0.636–0.706), with an overall accuracy of 0.883. Despite the recall being lower at 0.177, XGBoost outperformed all the other models when it came to AUC for this period. For 6-month predictions, XGBoost once again achieved the highest AUC (0.678, 95% CI: 0.638–0.718). It also had a better F1-score (0.357) and recall (0.349) when compared against every other algorithm the study tested. For the 12-month predictions, though XGBoost produced an AUC of 0.644 (95% CI: 0.589–0.699)—this number was slightly lower than the AUC that Random Forest got, which was 0.646. However, Random Forest showed a notably low recall of just 0.160, and its F1-score was only 0.206. By comparison, XGBoost achieved a recall of 0.510 and an F1-score of 0.318, which shows it has a favorable balance between sensitivity and precision.

### 3.4. Final Model Interpretation

**Figure 3:**
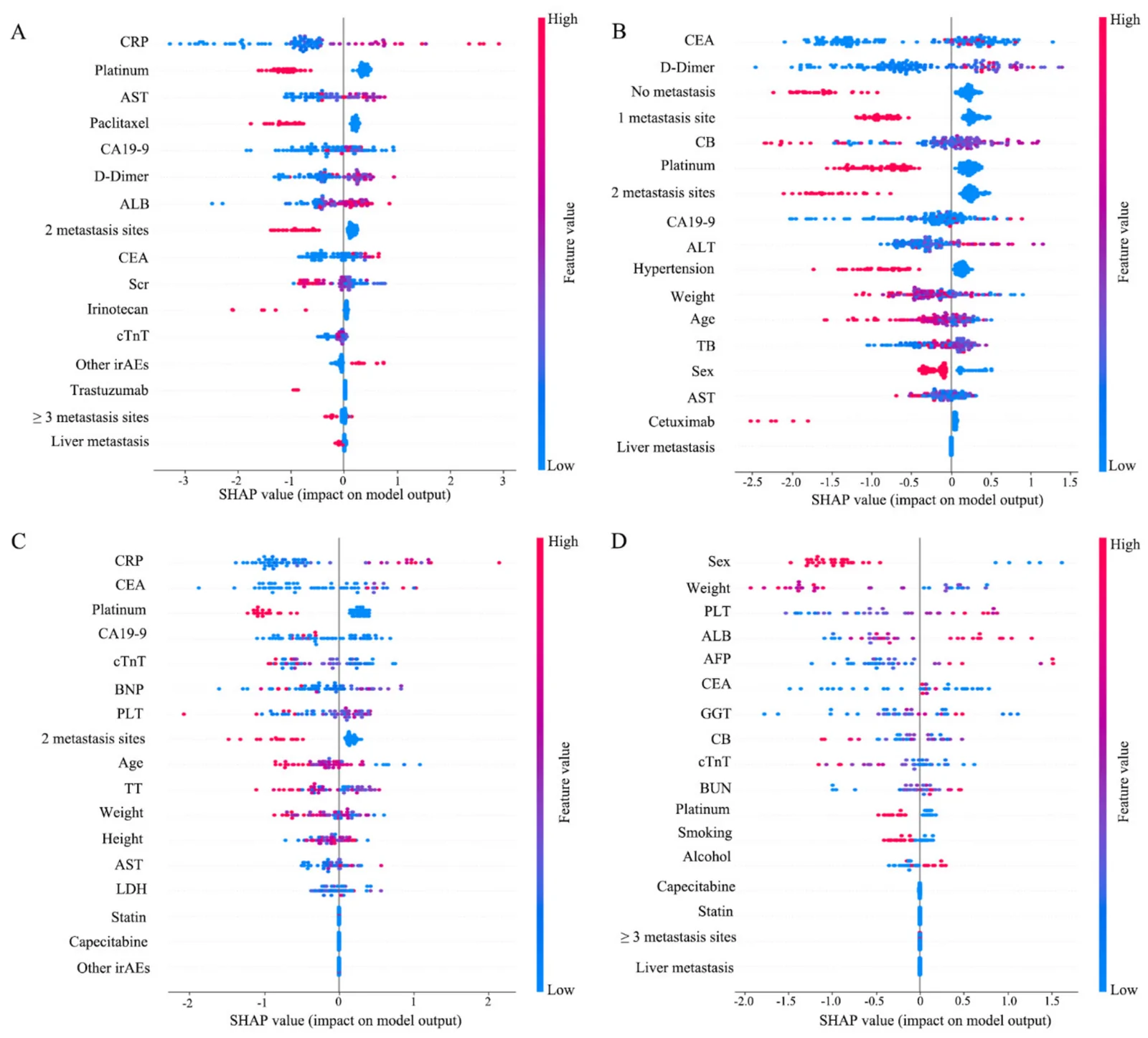
SHAP explanation for the final model. (**A**) SHAP summary for the Gradient Boost model of ICILI ≥ grade 2. (**B**) SHAP summary for the XGBoost model in the 3-month prediction. (**C**) SHAP summary for the XGBoost model in the 6-month prediction. (**D**) SHAP summary for the XGBoost model in the 12-month prediction. SHAP: Shapley Additive exPlanations; ICILI: Immune checkpoint inhibitor-associated liver injury; CRP: C-reactive protein; CA19-9: carbohydrate antigen 19-9; AST: aspartate aminotransferase; ALT: alanine aminotransferase; CEA: carcinoembryonic antigen; cTnT: cardiac troponin T; BNP: B-type Natriuretic Peptide; Scr: serum creatinine; ALB: albumin; TB: Total Bilirubin; AFP: Alpha-fetoprotein; PLT: Platelet; irAEs: immune-related adverse event.

The SHAP was utilized to interpret how the final model works and provide a comprehensive understanding at four time points (Figure 3). In the prediction of ICILI ≥ grade 2 (Figure 3A), 3 features that affected the output of the GradientBoost model were baseline CRP, platinum-based agents and baseline AST. Higher levels (marked in red) of both CRP and AST were closely associated with ICILI incidence, whereas ICI therapy based on platinum agents exerted a negative effect.

For the 3-month prediction model (Figure 3B), CEA and D-dimer turned out to be the primary determinants. Both higher and lower CEA levels exhibited a significant bidirectional effect at 3-month follow-up. In the 6-month model (Figure 3C), CRP remained the principal predictor, whereas the importance of features such as BNP, PLT, and the number of metastatic lesions increased substantially compared with the model of ICILI ≥ grade 2.

In the 12-month prediction (Figure 3D), sex and body weight were identified as the most important features. According to Figure 3D, male and lower body weight significantly increased the ICILI. Such results might suggest that host factors such as gender and nutritional status play a progressively pivotal role in susceptibility to liver injury during prolonged immunotherapy.

### 3.5. Sensitivity Analyses for ICILI Attribution

To tackle the issue that patients with RUCAM ≥ 3 may encompass diagnostically ambiguous events, and to assess if the prediction model was predominantly influenced by confounding variables like liver metastases or concurrent anticancer treatments, we conducted a series of sensitivity analyses. As shown in Supplementary Table S4, the GradientBoost model yielded an AUC of 0.781 (95% CI: 0.745–0.817), which is similar to the model of ICILI ≥ grade 2 (AUC = 0.769). The accuracy, precision, and Recall were recorded as 0.862, 0.288, and 0.348, respectively, compared to 0.834, 0.266, and 0.360 in the model of ICILI ≥ grade 2. Time-sensitive XGBoost models conforming to the rigorous criteria attained AUCs of 0.683 at 3 months, 0.651 at 6 months, and 0.659 at 12 months, consistent with the principal findings.

To assess if the model’s prediction efficacy was influenced by liver metastases or concomitant TKIs, we conducted subgroup analyzes omitting individuals with these characteristics (Supplementary Table S5). In patients without liver metastases (n = 947, events = 67), the GradientBoost model obtained an AUC of 0.752 (95% CI: 0.711–0.793), with an accuracy of 0.849, a precision of 0.255, and a recall of 0.345. Time-sensitive XGBoost models within this subgroup yielded AUCs of 0.658 for 3-month, 0.635 at 6-month, and 0.638 for 12-month, all of which were consistent with the performance of the overall cohort. In the sample that did not include patients on concomitant TKIs (n = 1277, events = 108), the GradientBoost model exhibited strong performance with an AUC of 0.764 (95% CI: 0.727–0.801), an accuracy of 0.852, a precision of 0.263, and a recall of 0.355. The AUCs for time-specific XGBoost were 0.669, 0.641, and 0.643 at 3, 6, and 12 months, respectively.

## 4. Discussion

The study established and validated prediction models for grade ≥ 2 ICILI at specific time points in patients with GC. By applying five ML algorithms with the SHAP method, our models achieved predictive performance, with the highest AUC of 0.769 for grade ≥2 ICILI in the model of GradientBoost.

ICILI is identified through abnormal laboratory test results, with reported incidence ranging from 1% to 15% in randomized trials [21,22,23]. Among patients with GC receiving ICIs, the incidence of any grade of irAEs ranged from 21.2% to 48.7%, while the hepatotoxicity rates ranged from 13.2% to 17.5%, and ICILI of grade 2 was 11% [24,25,26]. In the present study, the prevalence of any grade of ICILI was 13.5%, consistent with reported ranges.

Previous models for ICILI have predominantly depended on baseline characteristics [15,16]; however, the predictive factors may vary across treatment cycles, and there is a deficiency of multi-timepoint models. To address this gap, the current research developed predictive stratification tools at designated intervals throughout ICI therapy. The GradientBoost model for grade ≥ 2 ICILI yielded an AUC of 0.769, similar to the findings of Jun et al. [16]; nevertheless, XGBoost models at specific time points produced AUCs ranging from 0.644 to 0.678. Our models improve current static models by providing timepoint-specific predictive estimates, thereby broadening the evidence foundation for ICILI risk classification.

The final GradientBoost model integrated multiple features, in which baseline CRP, alongside platinum and AST, appeared as predictive features for predicting ICILI grade ≥ 2. Such a combination provides insight into the inflammatory mechanisms that trigger ICILI. As an acute-phase protein, CRP is correlated with systemic inflammation and reflects the activation of pro-inflammatory cytokines such as interleukin-6 (IL-6) [27,28]. Elevated AST levels directly reflect the degree of hepatocyte injury, and previous research has shown that increased baseline AST/ALT levels and baseline CRP ≥ 8.2 mg/L are significantly correlated with grade 3 or higher ICILI [29,30]. The features contributing to model predictions in short-term prediction indicated that ICILI reflects an acute liver injury event initiated by ICI-induced deterioration of immune tolerance amongst pre-existing inflammation.

The XGBoost model showed an improvement in the relevance of D-dimer and carcinoembryonic antigen (CEA) at the 3-month prediction interval. The occurrence of irAEs involves T-cells targeting hepatocytes, followed by tissue repair processes where coagulation plays a key role [31]. Research on single-cell has demonstrated that during ICILI, gene pathways associated with oxidative stress and pyroptosis exhibited elevated activity in particular blood cells. Additionally, T-cell receptor diversity was increased, indicating an association between T-cell proliferation and tissue injury [32]. Increased CEA levels, which are associated with tumor burden, indicate a greater tumor antigen load. This could result in enhanced T-cell activity and a raised likelihood of irAEs [33], as confirmed by the midterm predictors established in the model.

The features of BNP and platelet counts contributing to the model development in the 6-month suggested that ICILI extended beyond hepatic symptoms. Cancer patients receiving ICIs therapy frequently encounter an escalating risk of multisystem irAEs, including cardiovascular complications [34]. The present study revealed that BNP levels increase during mid-term prediction, suggesting that immune-related cardiomyopathy and liver injury may have a shared immunopathological basis, both driven by the aberrant activation of CD8^+^ T cells [35].

In the 12-month long-term model, gender and BMI were predictive features, which differed from earlier studies [14,23,36,37]. Previous studies have indicated that females were at a greater risk of experiencing grade 3 or higher ICILI compared to males [30,38]. However, a multivariable analysis of a retrospective study involving 1096 patients treated with ICI found that gender was not a significant predictor [39]. The reasons for this disparity, whether due to differences in outcome definitions or variations in ICI pharmacokinetics and pharmacodynamics, remain unclear. As for BMI, the SHAP analysis in the present study indicated that a low BMI associated with model-estimated risk, consistent with previous evidence that extreme BMI levels, specifically underweight and obesity, are associated with a higher risk of liver damage [40]. However, a pooled analysis showed that patients with BMI ≥ 30 kg/m^2^ had an increased risk of irAEs compared to those with normal BMI [41], a finding supported by a longitudinal cohort study [11]. Therefore, low and high BMI may influence medication pharmacokinetics, including absorption, distribution, metabolism, and excretion [42,43].

Notably, statins were identified as a key variable in the prediction model at both 6 and 12 months, indicating that concurrent drugs with immunomodulatory or hepatotoxic effects might affect the possibility of ICILI. This observation is consistent with growing evidence that commonly prescribed medications, such as proton pump inhibitors (PPIs) and antibiotics, can influence gut microbiota composition or systemic inflammation [44,45]. A combination of statins in our model may indicate analogous immunomodulatory pathways, although the precise mechanism remains unclear. Nonetheless, since this retrospective analysis applied electronic medical data, we could not fully identify all potentially relevant concomitant drugs. Future investigations with comprehensive medication analysis are essential to elucidate the influence of concomitant drugs on ICILI progression.

Several limitations must be acknowledged. Initially, while we evaluated potential survivor bias by comparing characteristics between retained and censored patients and found that most clinical parameters were comparable across groups, we cannot entirely eliminate residual confounding from other variables, such as treatment regimens or cumulative drug exposure, which may have affected both patient retention and ICILI risk. In addition, the substantial reduction in sample size at 6 and 12 months could constrain the applicability of our models at later time points. Furthermore, our models were constructed only with baseline data and do not include longitudinal biomarkers. Thus, our approach should be regarded as time-stratified rather than genuinely dynamic prediction. Subsequent research integrating serial biomarker assessments into joint modeling, along with external validation in larger multicenter populations, is needed to corroborate our results.

In conclusion, this study developed time-stratified and interpretable ML models to predict grade ≥ 2 ICILI in patients with GC across multiple time intervals, overcoming the limitation of current single-time prediction models. SHAP-based analysis showed that predictive variables vary over time, from acute inflammatory indicators to host-related factors, giving clinicians insights for time-stratified monitoring during immunotherapy.

## Figures and Tables

**Table 1:** Baseline clinical characteristics of the study population at multiple intervals post-ICI treatment.

Characteristics	ICI Treatment Within 3 Months			ICI Treatment Within 6 Months			ICI Treatment Within 12 Months		
	Non-ICILI (N = 1225)	ICILI (N = 112)	*p*-Value	Non-ICILI (N = 743)	ICILI (N = 106)	*p*-Value	Non-ICILI (N = 353)	ICILI (N = 48)	*p*-Value
Age (Years)	65.00 (58.00, 71.00)	64 (53.25, 70.00)	0.455	65.00 (58.00, 71.00)	65.00 (52.75, 70.00)	0.649	66.00 (59.00, 71.50)	66.50 (60.00, 70.75)	0.921
Male, n (%)	915 (74.7%)	83 (74.1%)	0.485	558 (75.1%)	73 (68.9%)	0.169	269 (76.2%)	30 (62.5%)	0.041
BMI (kg/m^2^)	23.23 (21.17, 25.50)	23.29 (20.20, 25.72)	0.921	23.43 (21.26, 25.64)	22.80 (19.98, 25.31)	0.755	23.53 (21.35, 25.60)	23.46 (19.84, 25.72)	0.921
HBV infection, n (%)	58 (4.7%)	9 (8.0%)	0.100	37 (5.0%)	7 (6.6%)	0.480	23 (6.5%)	3 (6.3%)	0.944
Non-alcoholic fatty liver, n (%)	106 (8.7%)	14 (12.5%)	0.119	69 (9.3%)	7 (6.6%)	0.365	31 (8.8%)	4 (8.3%)	0.918
Diabetes, n (%)	158 (12.9%)	14 (12.5%)	0.904	104 (14.0%)	17 (16.0%)	0.574	45 (12.7%)	7 (14.6%)	0.722
Hypertension, n (%)	347 (28.3%)	31 (27.7%)	0.884	205 (27.6%)	31 (29.2%)	0.722	95 (26.9%)	14 (29.2%)	0.742
Liver metastasis, n (%)	338 (27.6%)	52 (46.4%)	0.000	218 (29.3%)	52 (49.1%)	0.000	103 (29.2%)	22 (45.8%)	0.019
**Number of tumor metastasis sites**									
1~2, n (%)	728 (59.4%)	70 (62.5%)	0.526	441 (59.35%)	68 (64.15%)	0.574	207 (58.6%)	32 (66.7%)	0.288
≥3, n (%)	131 (10.7%)	23 (20.5%)	0.002	81 (10.9%)	20 (18.9%)	0.018	38 (10.8%)	8 (16.7%)	0.229
**Baseline of laboratory parameters**									
RBC	4.11 (3.69, 4.48)	4.10 (3.75, 4.43)	0.665	3.68 (4.12, 4.48)	4.04 (3.71, 4.39)	0.434	4.07 (3.63, 4.46)	3.98 (3.69, 4.36)	0.798
Hb	125.00 (112.00, 136.00)	124.00 (110.25, 135.00)	0.004	125.00 (112.00, 137.00)	122.00 (109.00, 133.00)	0.186	125.00 (111.00, 137.00)	125.00 (113.25, 134.00)	0.853
PLT	208.50 (167.00, 236.25)	227.00 (173.00, 280.00)	0.080	209.00 (163.50, 264.50)	224.00 (175.00, 280.75)	0.214	201.00 (158.00, 261.00)	227.50 (179.50, 267.75)	0.040
WBC	5.76 (4.60, 7.17)	6.11 (4.68, 7.83)	0.084	5.80 (4.58, 7.18)	5.75 (4.68, 7.81)	0.927	5.61 (4.48, 6.97)	5.72 (4.84, 7.81)	0.631
TBIL (μmol/L)	8.60 (6.40, 11.80)	8.35 (6.02, 11.78)	0.882	6.3 (8.6, 11.8)	8.40 (6.60, 12.30)	0.856	8.80 (6.50, 12.65)	7.95 (6.60, 11.82)	0.209
DB (μmol/L)	2.40 (1.70, 3.50)	2.50 (1.72, 3.50)	0.189	2.40 (1.70, 3.40)	2.60 (1.78, 3.70)	0.350	2.50 (1.70, 3.60)	2.15 (1.70, 3.55)	0.442
ALT (U/L)	16.00 (11.00, 24.00)	21.50 (14.00, 35.75)	0.000	17.00 (12.00, 25.00)	21.50 (14.00, 37.25)	0.005	17.00 (16.00, 26.00)	25.00 (15.00, 39.50)	0.023
AST (U/L)	21.00 (16.00, 26.88)	25.50 (20.00, 36.00)	0.000	21.00 (16.00, 26.00)	25.00 (19.00, 36.25)	0.000	21.00 (16.00, 26.00)	29.50 (19.25, 46.00)	0.008
ALP (U/L)	87.00 (70.00, 107.00)	98.00 (74.25, 126.75)	0.000	87.00 (71.00, 107.00)	96.00 (72.00, 126.25)	0.199	88.00 (70.00, 108.00)	83.00 (70.50, 122.50)	0.951
GGT (U/L)	25.00 (17.00, 46.00)	37.00 (21.00, 90.00)	0.000	26.00 (17.00, 48.00)	34.00 (21.50, 75.50)	0.043	27.00 (17.00, 46.00)	34.00 (23.25, 66.00)	0.130
INR	1.03 (0.98, 1.07)	1.04 (0.99, 1.10)	0.540	1.03 (0.98, 1.07)	1.04 (0.99, 1.11)	0.452	1.03 (0.97, 1.08)	1.03 (0.97, 1.07)	0.872
CRP (mg/dL)	2.6 (0.8, 7.00)	11.45 (3.05, 40.13)	0.000	2.7 (0.9, 7.8)	11.40 (2.75, 32.18)	0.000	2.10 (1.00, 6.70)	10.50 (2.68, 35.17)	0.002
AFP	3.00 (2.20, 4.30)	3.10 (2.12, 4.98)	0.005	3.00 (2.20, 4.20)	3.15 (2.12, 5.07)	0.475	3.00 (2.10, 4.10)	3.25 (2.08, 5.70)	0.419
**Combination with targeted therapy**									
TKI	49 (5.7%)	11 (11.8%)	0.022	22 (9.3%)	9 (9.1%)	0.958	24 (6.8%)	4 (8.3%)	0.696
EGFR	23 (2.7%)	1 (1.1%)	0.347	2 (0.8%)	1 (1.1%)	0.806	7 (2.0%)	1 (2.1%)	0.963
VEGF	9 (1.1%)	0 (0.0%)	0.320	1 (0.4%)	0 (0.0%)	0.542	2 (0.6%)	1 (2.1%)	0.253

BMI: body mass index; RBC: red blood cells; Hb: hemoglobin; PLT: Platelet; WBC: white blood cells; TBIL: total bilirubin; DB: direct bilirubin; AST: aspartate aminotransferase; ALT: alanine aminotransferase; ALP: alkaline phosphatase; GGT: gamma- glutamyl transferase; INR: international normalized ratio; CRP: C-reactive protein; AFP: alpha-fetoprotein; TKI: tyrosine kinase inhibitor; EGFR: epidermal growth factor receptor; VEGF: vascular endothelial growth factor.

**Table 2:** Predictive performance of the machine learning models at distinct time points.

Model	AUC (95% CI)	Accuracy (95% CI)	Precision (95% CI)	Recall (95% CI)	F1-Score (95% CI)
**Predictive models for ICILI ≥ grade 2**					
LogisticRegression	0.759 (0.722, 0.796)	0.755 (0.739, 0.771)	0.242 (0.215, 0.269)	0.585 (0.517, 0.653)	0.340 (0.305, 0.375)
RandomForest	0.768 (0.745, 0.791)	0.855 (0.850, 0.860)	0.277 (0.202, 0.352)	0.322 (0.237, 0.407)	0.298 (0.378, 0.218)
AdaBoost	0.682 (0.637, 0.727)	0.824 (0.810, 0.838)	0.290 (0.256, 0.324)	0.396 (0.350, 0.442)	0.328 (0.297, 0.359)
GradientBoost	0.769 (0.732, 0.806)	0.834 (0.822, 0.846)	0.266 (0.193, 0.339)	0.360 (0.266, 0.454)	0.304 (0.223, 0.385)
XGBoost	0.768 (0.740, 0.796)	0.836 (0.825, 0.847)	0.274 (0.225, 0.323)	0.322 (0.259, 0.385)	0.292 (0.239, 0.345)
**Predictive models for ICILI ≥ grade 2 within 3 months**					
LogisticRegression	0.628 (0.590, 0.666)	0.812 (0.797, 0.827)	0.168 (0.138, 0.198)	0.310 (0.246, 0.374)	0.214 (0.177, 0.251)
RandomForest	0.663 (0.632, 0.694)	0.864 (0.872, 0.856)	0.230 (0.186, 0.274)	0.276 (0.216, 0.336)	0.246 (0.197, 0.295)
AdaBoost	0.636 (0.604, 0.658)	0.815 (0.799, 0.831)	0.179 (0.212, 0.146)	0.326 (0.265, 0.387)	0.228 (0.187, 0.269)
GradientBoost	0.630 (0.590, 0.670)	0.848 (0.838, 0.858)	0.180 (0.139, 0.221)	0.244 (0.175, 0.313)	0.205 (0.153, 0.257)
XGBoost	0.671 (0.636, 0.706)	0.883 (0.876, 0.890)	0.236 (0.208, 0.264)	0.177 (0.132, 0.222)	0.192 (0.153, 0.231)
**Predictive models for ICILI ≥ grade 2 within 6 months**					
LogisticRegression	0.591 (0.548, 0.634)	0.672 (0.643, 0.701)	0.231 (0.201, 0.261)	0.451 (0.406, 0.496)	0.303 (0.270, 0.336)
RandomForest	0.642 (0.586, 0.698)	0.771 (0.789, 0.817)	0.367 (0.331, 0.403)	0.287 (0.228, 0.346)	0.292 (0.245, 0.339)
AdaBoost	0.562 (0.504, 0.620)	0.749 (0.721, 0.777)	0.293 (0.246, 0.340)	0.371 (0.321, 0.421)	0.319 (0.280, 0.358)
GradientBoost	0.599 (0.559, 0.639)	0.771 (0.748, 0.794)	0.311 (0.269, 0.353)	0.349 (0.295, 0.403)	0.321 (0.279, 0.363)
XGBoost	0.678 (0.638, 0.718)	0.793 (0.779, 0.807)	0.317 (0.274, 0.360)	0.349 (0.318, 0.380)	0.357 (0.325, 0.389)
**Predictive models for ICILI ≥ grade 2 within 12 months**					
LogisticRegression	0.621 (0.543, 0.699)	0.802 (0.776, 0.828)	0.247 (0.176, 0.318)	0.280 (0.178, 0.382)	0.252 (0.172, 0.332)
RandomForest	0.646 (0.576, 0.725)	0.843 (0.829, 0.857)	0.367 (0.181, 0.558)	0.160 (0.085, 0.235)	0.206 (0.117, 0.307)
AdaBoost	0.643 (0.580, 0.706)	0.785 (0.765, 0.805)	0.167 (0.092, 0.242)	0.160 (0.085, 0.235)	0.159 (0.089, 0.230)
GradientBoost	0.609 (0.525, 0.693)	0.814 (0.788, 0.840)	0.350 (0.273, 0.463)	0.280 (0.200, 0.360)	0.306 (0.216, 0.396)
XGBoost	0.644 (0.589, 0.699)	0.704 (0.673, 0.735)	0.239 (0.199, 0.282)	0.510 (0.396, 0.624)	0.318 (0.262, 0.374)

## Data Availability

The original data in the study are available on request from the corresponding author.

## Supplementary Materials

The following supporting information can be downloaded at https://www.mdpi.com/article/10.3390/curroncol33090568/s1, Table S1: Characteristics of patients stratified by hepatotoxicity grade among individuals with gastrointestinal tumors. Table S2: Comparison of baseline characteristics between patients retained versus censored at 6-month and 12-month follow-up. Table S3: Feature selection summary for ICILI prediction models at each time point. Table S4: Model performance for patients with RUCAM ≥ 5 across multiple time points. Table S5: Subgroup analyses of model performance excluding patients with potential confounders. Figure S1. Feature selection results for ICILI risk prediction using LASSO and Random Forest across multiple time points.
