# Supplementary material for: Prediction of Immune Checkpoint Inhibitor-Induced Liver Injury in Patients with Gastrointestinal Cancer: Machine Learning Modeling for Time-Stratified Risk Assessment
Source: Curr Oncol. 2026 Sep 20;33(9):568. doi: 10.3390/curroncol33090568 (PMC13605956; doi:10.3390/curroncol33090568)

Supplementary Figure S1 Feature selection results for ICILI risk prediction using LASSO and Random Forest across multiple time points. (A-C) LASSO coefficient path, LASSO selected feature coefficients and Random Forest selected feature importances for comprehensive prediction. (D-F) LASSO coefficient path, LASSO selected feature coefficients and Random Forest selected feature importances for 3-month prediction. (G-I) LASSO coefficient path, LASSO selected feature coefficients and Random Forest selected feature importances for 6-month prediction. (J-L) LASSO coefficient path, LASSO selected feature coefficients and Random Forest selected feature importances for 6-month prediction. ICILI: Immune checkpoint inhibitor-associated liver injury; CRP: C-reactive protein; CA19-9: carbohydrate antigen 19-9; AST: aspartate aminotransferase; CEA: carcinoembryonic antigen; cTnT: cardiac troponin T; Scr: serum creatinine; ALB: albumin; irAEs: immune-related adverse events.

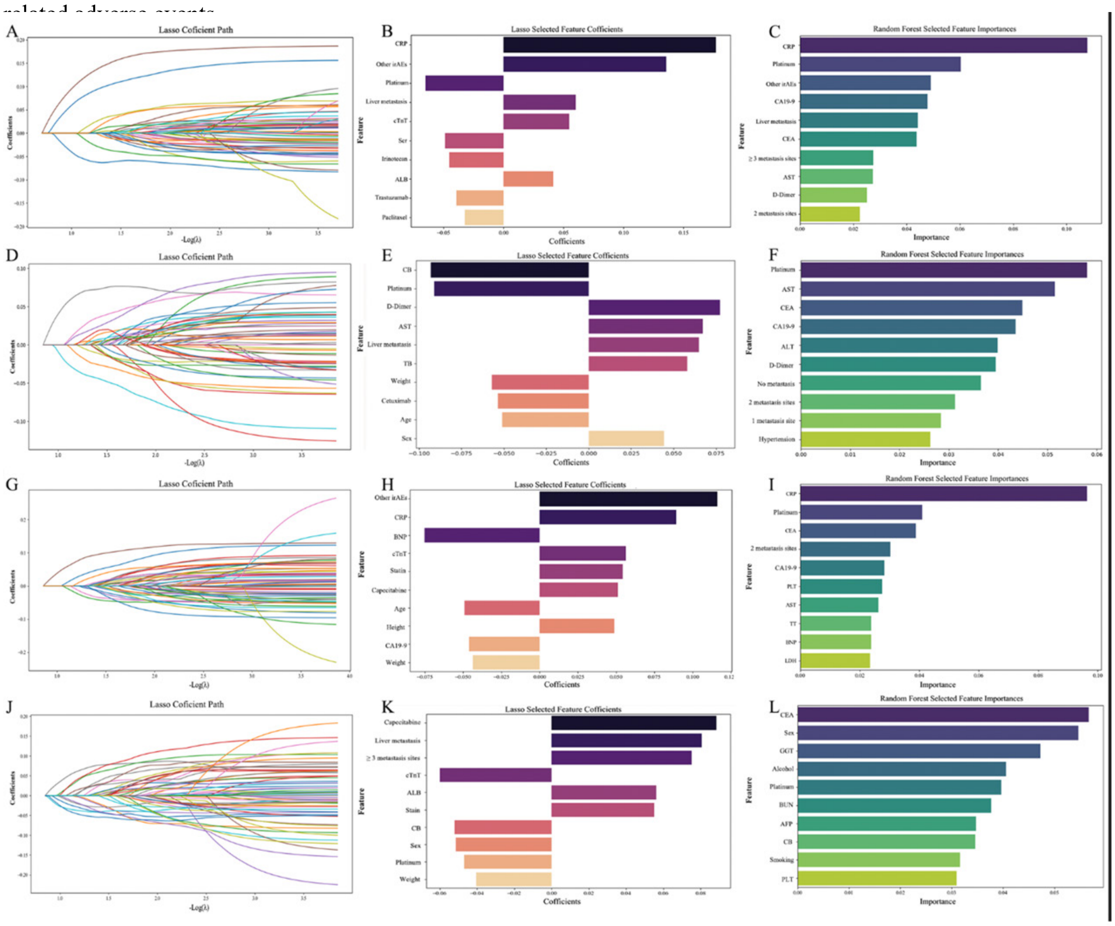

Supplement: Supplementary file 1 [file curroncol-33-00568-s001.zip › Supplementary Figure S1 Feature selection results for ICILI risk prediction using LASSO and Random Forest across multiple time points.pdf]
