# Supplementary material for: Prediction of Immune Checkpoint Inhibitor-Induced Liver Injury in Patients with Gastrointestinal Cancer: Machine Learning Modeling for Time-Stratified Risk Assessment
Source: Curr Oncol. 2026 Sep 20;33(9):568. doi: 10.3390/curroncol33090568 (PMC13605956; doi:10.3390/curroncol33090568)
Supplement: Supplementary file 1 [file curroncol-33-00568-s001.zip › supplementary Table S1.pdf]

Supplementary Table S1. Characteristics of patients stratified by hepatotoxicity grade among individuals with gastrointestinal tumors.

**Characteristic**	**Overall**<br>**N = 1337**	**Grade 1**<br>**N = 62, 4.6%**	**Grade 2**<br>**N = 54, 4.0%**	**Grade 3**<br>**N = 53, 3.9%**	**Grade 4**<br>**N = 12, 0.9%**	**P value**
Age (years) and Median (IQR)	65 (57 - 71)	62 (54.75 – 68.25)	63.50 (52.75 – 70.00)	65.00 (50.00 – 70.00)	63.00 (57.75 – 71.50)	0.198
Sex, n (% Male)	998 (74.6%)	49 (79.0%)	40 (74.1%)	35 (66.0%)	9 (75.0%)	0.603
BMI (kg/m^2), Median (IQR)	23.25 (21.10 – 25.52)	24.60 (20.74 – 26.10)	22.60 (20.11 – 25.40)	23.80 (21.60 – 25.66)	21.88 (20.80 – 24.36)	0.170
Liver metastases, n (%)	299 (25.9%)	24 (38.7%)	27 (50.0%)	37 (69.8%)	3 (25.0%)	< 0.000
Alcohol, n (%)	345 (29.8%)	24 (38.7%)	18 (33.3%)	11 (20.8%)	1 (8.3%)	0.117
**Underlying liver disease**						
Diabetes mellitus, n (%)	172 (12.9%)	9 (14.5%)	6 (11.1%)	7 (13.2%)	1 (8.3%)	0.971
Hypertension, n (%)	378 (28.3%)	17 (27.4%)	17 (31.5%)	16 (30.2%)	2 (16.7%)	0.879
HBV, n (%)	67 (5.0%)	3 (4.8%)	3 (5.6%)	5 (9.4%)	3 (25.0%)	0.013
Fatty liver disease, n (%)	120 (9.0%)	8 (12.9%)	9 (16.7%)	7 (13.2%)	2 (16.7%)	0.087
**Baseline biochemistries, Median (IQR)**						
AST (U/L)	21.00 (16.00 – 27.00)	21.00 (16.00 – 31.00)	27.00 (18.75 – 34.50)	29.5 (21.70 – 43.50)	24.00 (19.25 – 32.25)	< 0.000
ALT (U/L)	17.00 (12.00 – 25.00)	20.50 (14.00 – 30.25)	20.50 (11.00 – 34.50)	22.50 (16.00 – 40.25)	27.50 (14.00 – 38.75)	< 0.000
TBIL (μmol/L)	8.60 (6.40 – 11.80)	8.45 (6.25 – 12.85)	7.90 (5.77 – 11.32)	9.40 (7.30 – 12.32)	8.75 (7.55 – 14.85)	0.258
DB (μmol/L)	2.40 (1.70 – 3.50)	2.60 (1.70 – 3.60)	2.15 (1.50 – 3.52)	2.60 (1.90 – 4.00)	2.55 (2.10 – 3.87)	0.577
ALP (U/L)	87.00 (70.62 – 108.00)	87.50 (70.00 – 108.00)	89.50 (73.00 – 129.00)	98.00 (79.00 – 123.00)	105 (80.75 – 140.12)	0.021
GGT (U/L)	26.00 (17.00 –	34.50 (21.70 – 63.75)	38.00 (23.00 – 89.25)	51.50 (24.00 –	57.00 (26.00 –	< 0.000

	48.00)			119.50)	121.00)	
CRP (mg/L)	3.00 (1.10 – 7.73)	5.40 (0.90 – 15.33)	7.35 (2.33 – 27.73)	19.30 (3.05 – 39.70)	41.80 (12.55 – 73.53)	< 0.000
D-Dimer (mg/L)						< 0.000
CEA (ng/mL)	3.30 (2.00 – 8.60)	3.50 (1.80 – 15.05)	7.30 (2.82 – 36.47)	11.10 (2.375 – 102.10)	5.90 (5.37 – 26.52)	< 0.000
AFP (ng/ml)	3.00 (2.20 – 4.30)	3.05 (2.12 – 4.27)	3.70 (2.90 – 5.40)	2.90 (2.00 – 5.10)	2.65 (1.77 – 4.47)	0.021
CA19-9 (U/mL)	16.40 (8.40 – 48.05)	24.20 (9.15 – 190.00)	27.35 (10.00 – 276.00)	59.10 (15.80 – 422.50)	93.80 (24.40 – 196.50)	< 0.000
**ICIs therapy**						0.392
PD-1 mAb, n (%)	1288 (96.3%)	61 (98.4%)	51 (94.4%)	53 (100%)	11 (91.7%)	
PD-L1 mAb, n (%)	49 (3.7%)	1 (1.6%)	3 (5.6%)	0 (0.0%)	1 (8.3%)	
**Targeted therapy**						
TKI, n (%)	98 (7.3%)	0 (0.0%)	6 (11.1%)	8 (15.1%)	3 (25.0%)	0.002
Bevacizumab, n (%)	12 (0.9%)	1 (1.6%)	0 (0.0%)	1 (1.9%)	0 (0.0%)	0.818
**Site of metastasis**						
1	495 (37.0%)	16 (25.8%)	17 (31.5%)	15 (28.3%)	6 (50.0%)	0.122
2	303 (22.7%)	16 (25.8%)	17 (31.5%)	23 (43.4%)	1 (8.3%)	0.001
⩾3	154 (11.5%)	14 (22.6%)	10 (18.5%)	11 (20.8%)	2 (16.7%)	0.002
Other irAEs, n (%)	108 (8.1%)	9 (14.5%)	10 (18.5%)	20 (37.7%)	4 (33.3%)	< 0.000
Cycles of the 1st ICI infusion to ICILI, Median (IQR)		4 (2 – 9)	4 (2 – 7)	5 (2 – 7)	2 (1 – 3)	0.245
Latent period, Median (IQR)		72.00 (34.00 – 146.00)	78.50 (43.25 – 130.50)	86.50 (50.75 – 150.50)	46.50 (23.75 – 76.00)	0.157

IQR: interquartile range; BMI: body mass index; HBV: hepatitis B virus; AST: aspartate aminotransferase; ALT: alanine aminotransferase; TBIL: total bilirubin; DB: direct bilirubin; ALP: alkaline phosphatase; GGT: gamma-glutamyltransferase; CRP: C-reactive protein; CEA: carcinoembryonic antigen; AFP: alpha-fetoprotein; CA19-9: carbohydrate antigen 19-9; PD-1: programmed cell death 1; PD-L1: programmed death-ligand 1;TKI: tyrosine kinase inhibitors; ICI: Immune checkpoint inhibitor; ICILI: ICI-related liver injury.
