# Supplementary material for: Prediction of Immune Checkpoint Inhibitor-Induced Liver Injury in Patients with Gastrointestinal Cancer: Machine Learning Modeling for Time-Stratified Risk Assessment
Source: Curr Oncol. 2026 Sep 20;33(9):568. doi: 10.3390/curroncol33090568 (PMC13605956; doi:10.3390/curroncol33090568)
Supplement: Supplementary file 1 [file curroncol-33-00568-s001.zip › Supplementary Table S2.pdf]

Supplementary Table S2. Comparison of baseline characteristics between patients retained versus censored at 6-month and 12-month follow-up

Characteristics	6-Month follow-up			12-Month follow-up		
	Retained (N = 849)	Censored (N = 488)	P-value	Retained (N = 401)	Censored (N = 936)	P-value
Age, median (IQR), years	65.0 (58.0, 71.0)	64.0 (57.0, 70.0)	0.240	66.0 (59.0, 71.0)	64.0 (57.0, 70.0)	0.062
Male, n (%)	631 (74.3%)	367 (75.2%)	0.721	299 (74.6%)	699 (74.7%)	0.964
BMI, median (IQR), kg/m²	23.42 (21.10, 25.60)	23.1 (21.1, 25.3)	0.098	23.6 (21.3, 25.7)	23.1 (21.0, 25.3)	0.053
Liver metastasis, n (%)	270 (31.8%)	120 (24.6%)	0.005	125 (31.2%)	265 (28.3%)	0.292
≥3 metastatic sites, n (%)	101 (11.9%)	53 (10.9%)	0.568	46 (11.5%)	108 (11.5%)	0.972
Baseline CRP, median (IQR), mg/dL	5.4 (0.8, 34.0)	5.7 (0.9, 30.1)	0.146	5.2 (1.2, 32.8)	5.4 (1.0, 33.6)	0.468
Baseline ALT, median (IQR), U/L	17.0 (16.0, 27.0)	16.0 (11.0, 24.0)	0.240	17.0 (12.0, 27.0)	16.0 (11.0, 24.0)	0.145
Baseline AST, median (IQR), U/L	21.0 (16.0, 27.0)	20.0 (16.0, 27.0)	0.188	22.0 (16.0, 28.0)	21.0 (16.0, 27.0)	0.043
Baseline ALP, median (IQR), U/L	88.0 (71.0, 108.7)	87.0 (69.0, 106.0)	0.101	88.0 (70.0, 108.2)	87.0 (71.0, 108.0)	0.661
Baseline GGT, median (IQR), U/L	27.0 (17.0, 50.0)	24.0 (16.0, 45.0)	0.296	28.0 (17.5, 48.0)	25.0 (17.0, 49.0)	0.580
Baseline PLT, median (IQR), ×10⁹/L	211.0 (166.5, 266.0)	208.0 (168.0, 263.7)	0.546	203.0 (160.0, 264.0)	212.0 (168.0, 266.2)	0.338
Baseline TBIL, median (IQR), μmol/L	8.6 (6.4, 11.8)	8.6 (6.5, 11.8)	0.365	8.7 (6.5, 12.3)	8.6 (6.3, 11.7)	0.297
Hypertension, n (%)	236 (27.8%)	142 (29.1%)	0.611	109 (27.2%)	269 (28.7%)	0.562
Diabetes mellitus, n (%)	121 (14.3%)	51 (10.5%)	0.056	52 (13.0%)	120 (12.8%)	0.941

BNP	70.2 (35.5, 128.7)	72.7 (39.0, 136.6)	0.896	73.5 (40.1, 134.5)	70.1 (36.4, 133.2)	0.105
Fatty liver disease, n (%)	76 (9.0%)	44 (9.0%)	0.968	35 (8.7%)	85 (9.1%)	0.836
HBV infection, n (%)	44 (5.2%)	23 (4.7%)	0.705	26 (6.5%)	41 (4.4%)	0.106
Platinum-based chemotherapy, n (%)	311 (36.6%)	202 (41.4%)	0.085	149 (37.2%)	364 (38.9%)	0.551
Concurrent TKIs, n (%)	31 (3.6%)	29 (5.9%)	0.109	28 (7.0%)	32 (3.4%)	0.000
