# Supplementary material for: Prediction of Immune Checkpoint Inhibitor-Induced Liver Injury in Patients with Gastrointestinal Cancer: Machine Learning Modeling for Time-Stratified Risk Assessment
Source: Curr Oncol. 2026 Sep 20;33(9):568. doi: 10.3390/curroncol33090568 (PMC13605956; doi:10.3390/curroncol33090568)
Supplement: Supplementary file 1 [file curroncol-33-00568-s001.zip › Supplementary Table S3.pdf]

Supplementary Table S3. Feature selection summary for ICILI prediction models at each time point

Time point	Top 10 variables by Random Forest importance	Variables retained by LASSO	Final predictor set	No. of predictors
Overall	CRP, Platinum, Other irAEs, CA19-9, Liver metastasis, CEA, ⩾3 metastasis sites, AST, D-Dimer, 2 metastasis sites	CRP, Other irAEs, platinum, Liver metastasis, cTnT, Scr, Irinotecan, ALB, Trastuzumab, Paclitaxel	CRP, Platinum, Other irAEs, CA19-9, Liver metastasis, ⩾3 metastasis sites, AST, D-Dimer, 2 metastasis sites, cTnT, Scr, Irinotecan, ALB, Trastuzumab, Paclitaxel	15
3-month	CB, Platinum, D-dimer, AST, Liver metastasis, TB, Weight, Capecitabine, Age, Sex	Platinum, AST, CEA, CA19-9, ALT, D-dimer, No metastasis, 2 metastasis sites, 1 metastasis site, Hypertension	CB, Platinum, D-dimer, AST, Liver metastasis, TB, Weight, Capecitabine, Age, Sex, CEA, CA19-9, ALT, No metastasis, 2 metastasis sites, 1 metastasis site, Hypertension	17
6-month	Other irAEs, CRP, BNP, cTnT, Stain, Capecitabine, Age, Height, CA19-9, Weight	CRP, Platinum, CEA, 2 metastasis, CA19-9, Platelet, AST, TT, BNP, LDH	Other irAEs, CRP, BNP, cTnT, Stain, Capecitabine, Age, Height, Weight, Platinum, CEA, 2 metastasis, CA19-9, Platelet, AST, TT, BNP, LDH	18
12-month	Capecitabine, Liver metastasis, ⩾3 metastasis sites, cTnT, ALB, Stain, CB, Scr, Platinum, Weight	CEA, Scr, GGT, Alcohol, Platinum, BUN, AFP, CB, Smoking, PLT	Capecitabine, Liver metastasis, ⩾3 metastasis sites, cTnT, ALB, Stain, CB, Scr, Platinum, Weight, CEA, Scr, GGT, Alcohol, Platinum, BUN, AFP, Smoking, PLT	19

AFP, alpha-fetoprotein; ALB, albumin; ALT, alanine aminotransferase; AST, aspartate aminotransferase; BNP, B-type natriuretic peptide; BUN, blood urea nitrogen; CA19-9, carbohydrate antigen 19-9; CB, carboplatin (or platinum-based regimen); CEA, carcinoembryonic antigen; CRP, C-reactive protein; cTnT, cardiac troponin T; GGT, gamma-glutamyl transferase; LDH, lactate dehydrogenase; PLT, platelet; Scr, serum creatinine; TB, total bilirubin; TT, thrombin time.
