# Supplementary material for: Prediction of Immune Checkpoint Inhibitor-Induced Liver Injury in Patients with Gastrointestinal Cancer: Machine Learning Modeling for Time-Stratified Risk Assessment
Source: Curr Oncol. 2026 Sep 20;33(9):568. doi: 10.3390/curroncol33090568 (PMC13605956; doi:10.3390/curroncol33090568)
Supplement: Supplementary file 1 [file curroncol-33-00568-s001.zip › Supplementary Table S4.pdf]

Supplementary Table S4. Model performance for patients with RUCAM ≥ 5 across multiple time points

Time point	Model	N	AUC (95% CI)	Accuracy (95% CI)	Precision (95% CI)	Recall (95% CI)	F1-score (95% CI)
Overall	GradientBoost	1337 (78)	0.781 (0.745–0.817)	0.862 (0.857–0.867)	0.288 (0.195–0.381)	0.348 (0.255–0.441)	0.316 (0.231–0.401)
3-month	XGBoost	1337 (71)	0.683 (0.649–0.717)	0.891 (0.884–0.898)	0.182 (0.136–0.228)	0.182 (0.136–0.228)	0.198 (0.149–0.247)
6-month	XGBoost	849 (68)	0.651 (0.595–0.707)	0.793 (0.779–0.807)	0.332 (0.276–0.388)	0.321 (0.276–0.366)	0.348 (0.316–0.380)
12-month	XGBoost	401 (31)	0.659 (0.590–0.728)	0.815 (0.792–0.838)	0.276 (0.183–0.369)	0.297 (0.198–0.396)	0.284 (0.194–0.374)
