# Supplementary material for: Prediction of Immune Checkpoint Inhibitor-Induced Liver Injury in Patients with Gastrointestinal Cancer: Machine Learning Modeling for Time-Stratified Risk Assessment
Source: Curr Oncol. 2026 Sep 20;33(9):568. doi: 10.3390/curroncol33090568 (PMC13605956; doi:10.3390/curroncol33090568)
Supplement: Supplementary file 1 [file curroncol-33-00568-s001.zip › Supplementary Table S5.pdf]

Supplementary Table S5. Subgroup analyses of model performance excluding patients with potential confounders

Time point	Model	N (events)	AUC (95% CI)	Accuracy (95% CI)	Precision (95% CI)	Recall (95 % CI)	F1-score (95% CI)
**Without liver metastases**							
Overall	GradientBoost	947 (67)	0.752 (0.711–0.793)	0.849 (0.843–0.855)	0.255 (0.170–0.340)	0.345 (0.248–0.442)	0.293 (0.211–0.375)
3-month	XGBoost	947 (60)	0.658 (0.619–0.697)	0.879 (0.872–0.886)	0.228 (0.183–0.273)	0.168 (0.121–0.215)	0.193 (0.151–0.235)
6-month	XGBoost	579 (54)	0.635 (0.574–0.696)	0.772 (0.756–0.788)	0.308 (0.248–0.368)	0.335 (0.290–0.380)	0.321 (0.283–0.359)
12-month	XGBoost	276 (26)	0.638 (0.579–0.697)	0.808 (0.785–0.831)	0.258 (0.166–0.350)	0.285 (0.186–0.384)	0.271 (0.178–0.364)
**Without TKIs**							
Overall	GradientBoost	1277 (108)	0.764 (0.727–0.801)	0.852 (0.846–0.858)	0.263 (0.192–0.334)	0.355 (0.262–0.448)	0.302 (0.220–0.384)
3-month	XGBoost	1277 (101)	0.669 (0.634–0.704)	0.882 (0.875–0.889)	0.235 (0.207–0.263)	0.175 (0.128–0.222)	0.198 (0.157–0.239)
6-month	XGBoost	818 (97)	0.641 (0.585–0.697)	0.770 (0.754–0.786)	0.314 (0.271–0.357)	0.347 (0.302–0.392)	0.329 (0.293–0.365)
12-month	XGBoost	373 (44)	0.643 (0.584–0.702)	0.806 (0.783–0.829)	0.241 (0.199–0.283)	0.305 (0.206–0.404)	0.269 (0.185–0.353)

AUC, area under the receiver operating characteristic curve; CI, confidence interval; TKI, tyrosine kinase inhibitor; ICILI, immune checkpoint inhibitor-related liver injury.
